# Coil-Only High-Frequency Lamb Wave Generation in Nickel Sheets

**DOI:** 10.3390/s24227141

**Published:** 2024-11-06

**Authors:** Yini Song, Yihua Kang, Kai Wang, Yizhou Guo, Jun Tu, Bo Feng

**Affiliations:** 1 School of Mechanical Science and Engineering, Huazhong University of Science and Technology, Wuhan 430074, China; songyini@hust.edu.cn (Y.S.); yihuakang@hust.edu.cn (Y.K.); wang2kai@hust.edu.cn (K.W.); m202270767@hust.edu.cn (Y.G.); 2 School of Mechanical Engineering, Hubei University of Technology, Wuhan 430068, China; juntu@hbut.edu.cn

**Keywords:** magnetostrictive guided wave, lamb wave, coil-only EMAT, nickel sheet

## Abstract

This study presents a novel, coil-only magnetostrictive ultrasonic detection method that operates effectively without permanent magnets, introducing a simpler alternative to conventional designs. The system configuration is streamlined, consisting of a single meander coil, an excitation source, and a nickel sheet, with both the bias magnetic field and ultrasonic excitation achieved by a composite excitation containing both DC and AC components. This design offers significant advantages, enabling high-frequency Lamb wave generation in nickel sheets for ultrasonic detection while reducing device complexity. Experimental validation demonstrated that an S0-mode Lamb wave at a frequency of 2.625 MHz could be effectively excited in a 0.2 mm nickel sheet using a double-layer meander coil. The experimentally measured wave velocity was 4.9946 m/s, with a deviation of only 0.4985% from the theoretical value, confirming the accuracy of the method. Additionally, this work provides a theoretical basis for future development of flexible MEMS-based magnetostrictive ultrasonic transducers, expanding the potential for miniaturized magnetostrictive patch transducers.

## 1. Introduction

Ultrasound can be generated using electromagnetic methods through two main mechanisms: the Lorentz force and the magnetostrictive force. In conventional electromagnetic ultrasound, eddy currents are induced in a metallic conductor within an alternating magnetic field, and a Lorentz force acts on the conductor, producing alternating stress that generates mechanical waves. When these waves fall within the ultrasonic frequency range, ultrasound is produced. Guo [1] focused on enhancing ultrasonic Lamb wave generation by designing an EMAT with racetrack coils and periodic permanent magnets, which boosts wave amplitude through a carefully aligned Lorentz-force mechanism, especially in non-ferromagnetic plates. Lorentz forces arise in non-ferromagnetic conducting materials when exposed to an applied bias and alternating magnetic fields. In contrast, ferromagnetic materials experience both Lorentz forces and magnetostrictive effects, with magnetostriction playing the dominant role in the transmission of ultrasonic guided waves [2,3].

Ultrasound research is typically categorized into bulk waves and guided waves. An important subtype of guided waves is Lamb waves, which are associated with thin plate or membrane materials and exhibit complex propagation patterns. Lamb waves are highly sensitive to both internal and surface flaws, as they can propagate over long distances in plate structures with minimal attenuation. Defects in these structures can be detected through Lamb wave reflection, and the time-of-flight (ToF) method can be used to determine the trajectory or precise location of the flaws [4].

Electromagnetic ultrasonic transducers (EMATs) are available in various configurations, typically requiring the use of permanent magnets. Joo Kyung Lee [5] generated omnidirectional Lamb waves (OLW) based on the magnetostrictive principle by positioning a permanent magnet at the center with a surrounding circular coil. Similarly, Sun [6] generated antisymmetric (A0) omnidirectional Lamb waves through a structure analogous to the aforementioned one but based on the electromagnetic actuator (EMAT) transduction principle of the Lorentz force mechanism.

In the absence of a biasing field, magnetostrictive materials display a relatively weak magnetostrictive effect. The application of a suitably biased magnetic field results in the rearrangement of the magnetic domains of these materials, thereby enhancing the efficiency and sensitivity of the transducer. Magnetostrictive patch transducers (MPTs) operate by utilizing the magnetostrictive effect, where an applied magnetic field induces mechanical strain in a ferromagnetic sheet patch, enabling the detection of stress changes or vibrations within the stuck and tested object. Liu [7] introduced a double-turn coil omnidirectional shear-horizontal wave magnetostrictive patch transducer (DC-OSH-MPT) array for imaging inspection of composite plates. In 1999, Riichi Murayama [8] studied the mechanism of an EMAT using the magnetostrictive effect to generate Lamb waves in thin steel sheets, finding that different driving mechanisms under and between the meander coil’s lead lines are key for accurately evaluating the drawability of the sheets. While these studies explored magnetostrictive material-generated ultrasound in plates, they generally operated within the kHz frequency range, limiting their suitability for high-speed, high-precision inspections. Generating a bias magnetic field in traditional electromagnetic ultrasound and magnetostrictive systems typically requires permanent magnets or electromagnets [9]. However, coil-only sensors offer advantages such as a compact design and high flexibility. In 2016, Rueter [10,11] first proposed the concept of a coil-only electromagnetic acoustic transducer and designed both the transducer and its excitation circuit. Park [12] advanced this work by employing a dual-coil configuration for magnetostrictive patch (MPT) detection, integrating a DC-biased magnetic field with high-frequency AC excitation. A novel coil-only magnetostrictive sensor structure was introduced, consisting of two coil layers—an outer DC-biased coil and an inner AC coil. However, Isla and Cegla [13] noted that mitigating crosstalk between multiple coils in such configurations can be challenging. As mentioned earlier, Magnetic Particle Testing (MPT) typically operates based on thin nickel sheets, magnets, and coils. However, placing magnets can be challenging in confined spaces, and coil-only configurations may lead to crosstalk issues.

The advancement of microelectromechanical systems (MEMS) has led to the development of micromachined ultrasonic transducers (MUTs), which are utilized in miniaturized ultrasound systems. Among these, piezoelectric micromachined ultrasonic transducers (PMUTs) offer enhanced capacitance and reduced electrical impedance, improving sensitivity by mitigating parasitic capacitance and facilitating integration with low-voltage electronics [14,15,16]. The development of micro-scale devices that operate on the magnetostrictive effect is still in its nascent stages and presents significant opportunities for exploration. Nickel, a naturally magnetostrictive material, serves as the core component in magnetostrictive transducers. Danial Gandomzadeh [17] developed tapered nickel cores as the central element of a magnetostrictive ultrasonic transducer. Nickel is also frequently used in MEMS processes due to its favorable properties [18,19,20]. The propagation of magnetic micromachined ultrasonic transducers (mMUTs) is proposed as a potential method for generating high-frequency Lamb waves in micro magnetostrictive sheets. Investigating the mechanisms for generating high-frequency ultrasound in nickel sheets is crucial for the development of mMUTs.

This study introduces an innovative coil-only magnetostrictive ultrasonic detection method that operates effectively without the use of permanent magnets. The system design is streamlined, consisting solely of a meander coil, an excitation source, and a nickel sheet. Both the bias magnetic field and the excitation and reception of ultrasonic waves are achieved by an excitation source, where a DC component current is combined with an AC component. This approach offers distinct advantages over traditional designs: it simplifies device architecture while enabling the generation of high-frequency Lamb waves for ultrasonic detection. Furthermore, this method introduces a novel configuration for magnetostrictive patch transducers and lays a theoretical foundation for the development of flexible MEMS-based magnetostrictive ultrasonic transducers.

## 2. Methodology

### 2.1. Magnetostrictive Constitutive Relation

**Figure 1:**
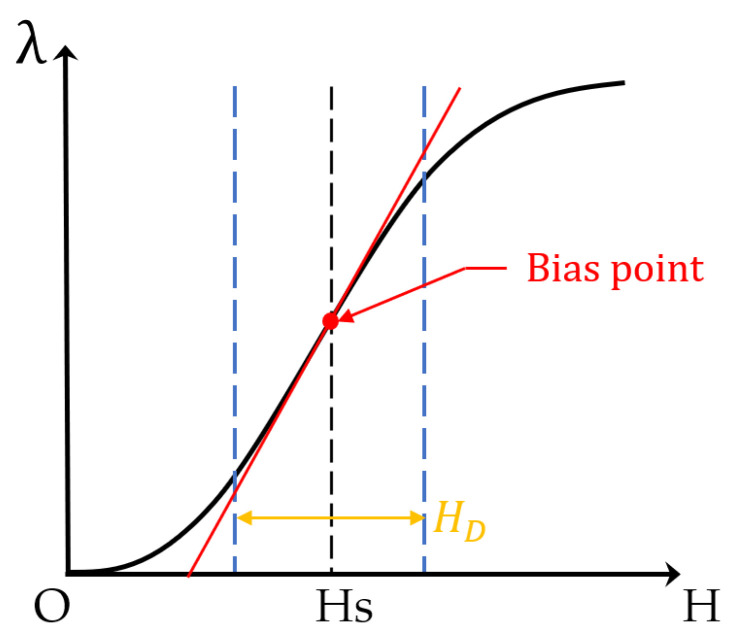
Magnetostriction curve.

Magnetostrictive elongation is generally nonlinear with respect to the applied magnetic field strength, and both the forward and reverse magnetostrictive effects exhibit this nonlinear behavior. The application of the Joule effect is one of the core principles behind many magnetostrictive devices, enabling the conversion of magnetic fields into mechanical motion or stress for sensing and actuation purposes, which typically requires the introduction of both a static bias field and a dynamic magnetic field. The use of an appropriate static bias field (*H_S_*) is crucial for the excitation of magnetostrictive phenomena and can be provided by a permanent magnet or an electromagnet. Once the bias magnetic field strength (*H_S_*) is applied, the introduction of a dynamic magnetic field *H_D_*) results in the oscillatory movement of the ferromagnetic material around the bias point in small amplitudes, generating a mechanical wave. In certain acoustic transducers operating at specific frequencies and under known conditions, a linear constitutive law can be used to simplify the material model. In practical magnetostrictive processes, this linear law is applied when *H_D_* << *H_S_*. Figure 1 shows a nonlinear magnetostriction curve, where *λ* represents the magnetostriction coefficient, and its magnitude equals the ratio of the elongation along the magnetization direction to the total length. The red dashed line in the figure represents the simplified linear magnetostriction effect at the bias point and can be interpreted as the instantaneous magnetostriction at that point.

A ferromagnetic material acts as both an elastomer and a piezomagnet or magnetostrictor. The magnetic field strength (*H*), magnetic induction strength (*B*), and magnetostrictive stress–strain can be described by the following Equation (1a,b), which are derived under the assumption that the ferromagnetic material operates with small oscillation amplitudes around the bias point, neglecting the effect of temperature.

(1a) $$\tilde{\varepsilon }=S^{H}\tilde{\sigma }+d\tilde{H}$$

(1b) $$\tilde{B}=d^{T}\tilde{\sigma }+\mu^{\sigma }\tilde{H}$$

In the above equations, *ε* and *σ* represent the strain and stress tensors, respectively.

*B* and *H* are the magnetic flux density and the magnetic field strength. $\tilde{\varepsilon },\;\tilde{\sigma },\;\tilde{B},\;and\;\tilde{H}$ are either directly or indirectly influenced by the *H_D_*, while $S^{H},\;d^{T},\;\;and\;\mu^{\sigma }$ are either directly or indirectly influenced by the *H_S_*. $S^{H}$ represents the elastic compliance matrix, which is measured when the magnetic field strength is constant. $\mu^{\sigma }$ represents the magnetic permeability matrix at constant stress and is determined from the nonlinear B–H curve, while it can be assumed that $S^{H}$ is not affected by the applied magnetic field. $S^{H}$, $\mu^{\sigma }$, and d can be affected by the applied static bias field.

**Figure 2:**
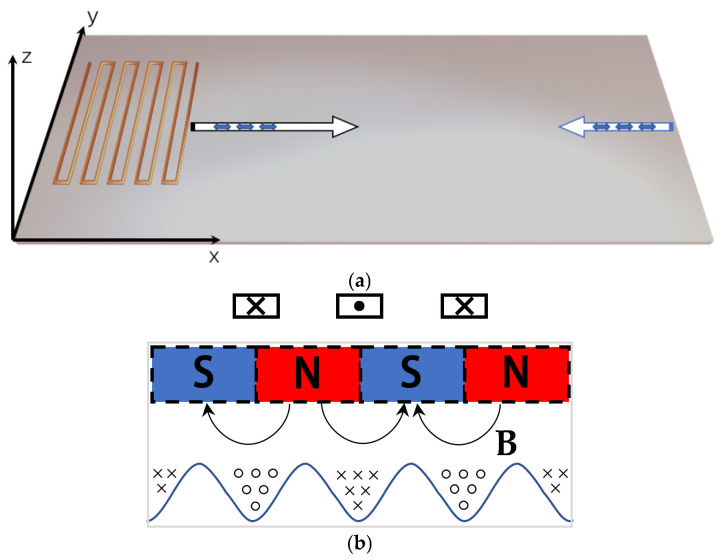
Working principle and schematic diagram: (**a**) Single-coil magnetostrictive ultrasonic transducer; (**b**) DC component through the coil generates a static magnetic field (*H_S_*), and the AC component produces a dynamic magnetic field (*H_D_*).

From the aforementioned fundamental principles of magnetostriction, it can be inferred that a static magnetic field is a prerequisite and that the static magnetic field is typically generated by an electromagnet or a permanent magnet. In the device illustrated in Figure 2a, however, neither permanent magnets nor electromagnets are employed to generate the bias magnetic field. The DC component of the signal passing through the meander coil is employed in the generation of *H_S_*. In accordance with Ampere’s law, the bias field generated by the aforementioned DC component in the direction of each wire in the vicinity of the nickel sheet can be equated to a plurality of N–S poles parallel to a strip of permanent magnets in the sheet (Figure 2b). The dynamic magnetic field *H_D_* is generated by the high frequency component.

In the current configuration, the orientations of both the dynamic and static magnetic fields are parallel to the x–z plane. The cross symbol is used to indicate that the magnetic field direction is from outside to inside, while the circle symbol represents a magnetic field directed from inside to outside. When these fields are parallel, no shear waves are generated in the sheet. At this point, the mass vibration direction of the Lamb wave lies in the x–y plane, and the vibration is parallel to the direction of ultrasonic propagation. Ideally, the Lamb wave in the S0 mode propagates along the x-direction and reflects upon reaching the end face. This will be described and analyzed in detail below.

The angle between the dynamic and static magnetic fields significantly influences the waveform modes. When the magnetic fields *H_S_* and *H_D_* are perpendicular to each other, a non-zero shear strain is generated in the magnetostrictive sheet, a phenomenon known as the Wiedemann effect. Conversely, when the *H_S_* is applied to the magnetostrictive sheet in a direction parallel to the *H_D_*, normal strain can be generated. If the angle between *H_D_* and *H_S_* is neither parallel nor perpendicular, both shear strain and normal strain will be generated. Figure 2b illustrates that both the dynamic and static magnetic fields are generated by the currents through the meander coil, which are parallel. This causes the nickel particle to move in the x-direction, resulting in the generation of a Lamb wave that propagates along the x-direction.

When the magnetic fields *H_S_* and *H_D_* act on a ferromagnetic sheet, they generate both a Lorentz force and a magnetostrictive force. The Lorentz force induces vibrations parallel to the thickness direction of the sheet and perpendicular to the vibrations generated by the magnetostrictive force.

### 2.2. Dispersion Diagram of Material

The cutoff frequency defines a boundary, below which Lamb waves with a finite number of modes can propagate at a given frequency. The cutoff frequency (*f_C_*) of a Lamb wave in a sheet is found to be closely related to the thickness of the sheet.

**Figure 3:**
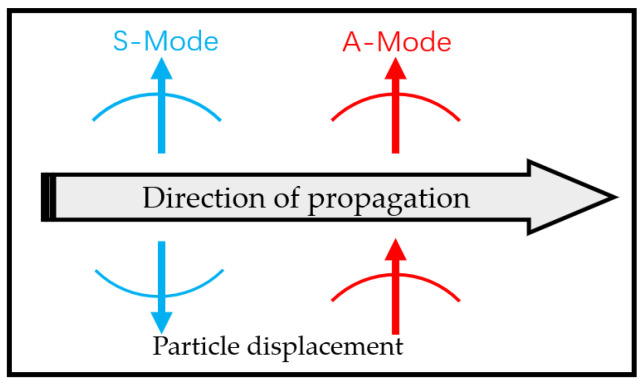
S-mode and A-mode Lamb wave propagation.

Lamb waves are categorized based on the distribution pattern of particle vibrational displacements. The two primary categories are symmetric Lamb wave (S-mode) and antisymmetric Lamb wave (A-mode), as shown in Figure 3. In the A mode, particle motion occurs transversely and exhibits an antisymmetric pattern, meaning that particles move in opposite directions on the upper and lower surfaces of the material. For the A0 mode specifically, particle motion is primarily an up-and-down shear, resembling a bending motion. The wave propagation direction for the A mode runs parallel to the surface of the plate. In the S mode, particle motion is symmetric, meaning particles move in the same direction on both the upper and lower surfaces of the material. Like the A mode, the propagation direction for the S mode also lies parallel to the plate surface. Within a given frequency range, which is influenced by the sheet’s thickness, different modes of Lamb waves (S0, S1, S2, etc.; A0, A1, A2, etc.) can propagate. Unlike SH waves, the 0th-order mode of Lamb waves is also dispersive. It is important to note the clear distinction between the particle vibration directions of A-mode and S-mode Lamb waves. In the case of S-mode Lamb waves, the particle vibration direction aligns with the propagation direction, as will be discussed later. This paper primarily focuses on the S0 mode of Lamb waves.

**Figure 4:**
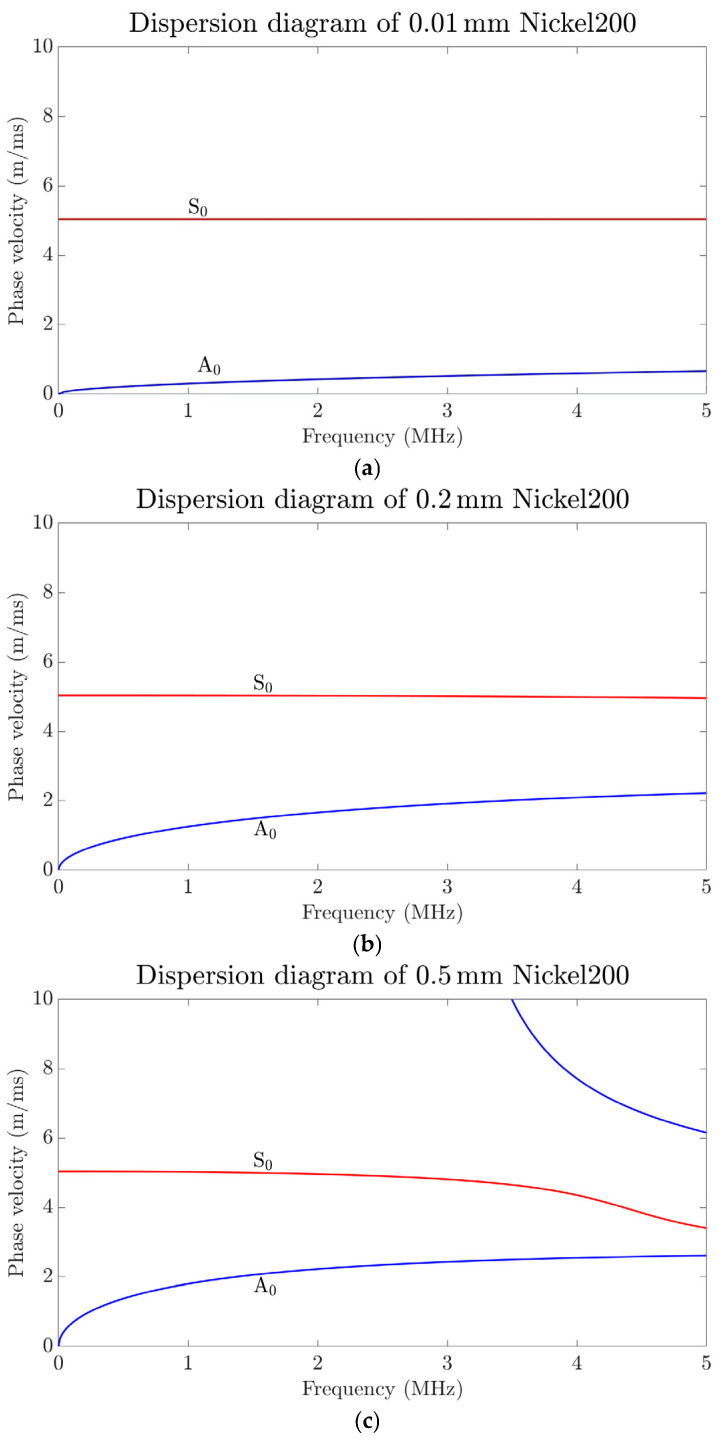
Lamb wave frequency dispersion diagrams for: (**a**) 0.01 mm, (**b**) 0.2 mm and (**c**) 0.5 mm nickel sheets.

The dispersion equation can be solved numerically based on wave speed, and the resulting dispersion curves are shown in Figure 4. The dispersion equation for Lamb waves derived from classical theory is expressed as follows [21]:

(2) $$\frac{\tan\left(k_{t}h\right)}{\tan\left(k_{l}h\right)}=-\frac{4k^{2}k_{l}k_{t}}{{\left(k^{2}-k_{t}^{2}\right)}^{2}}$$

(3) $$\frac{\tan\left(k_{t}h\right)}{\tan\left(k_{l}h\right)}=-\frac{{\left(k^{2}-k_{t}^{2}\right)}^{2}}{4k^{2}k_{l}k_{t}}$$

where $k_{l}=\omega^{2}/c_{l}^{2}-k^{2}$ and $k_{t}=\omega^{2}/c_{t}^{2}-k^{2}$ are wavenumbers of Lamb waves in the z direction. The symbols $c_{l}$ and $c_{t}$ denote the longitudinal and shear-wave speeds, respectively. In the case of symmetric Lamb waves, the most appropriate equation to employ is Equation (2). Conversely, for anti-symmetric Lamb waves, the equation utilized is Equation (3).

When the nickel sheet is sufficiently thin, pure A0 and S0 waves can be generated in the MHz range within a specific frequency band. The cutoff frequency (*f_C_*) determines how many waveform modes exist at a given frequency. For instance, *f_C-A1_* indicates that only zero-order Lamb waves are present when the actual frequency is below *f_C-A1_*. The cutoff frequencies can be found from the dispersion curves in Figure 4. As shown in Figure 4b above, *f_C-A1_* = 7.410 MHz when the thickness of the sheet is 0.2 mm, only two modes, S0 and A0, exist for the Lamb waves when the frequency is less than 7.410 MHz. Similarly, as shown in Figure 4c above, *f_C-A1_* = 2.964 MHz, meaning that only S0 and A0 modes exist below 2.964 MHz. When the sheet is sufficiently thin, pure zero-order waves can be generated within the 0 to 10 MHz range or even across a broader frequency spectrum. Additionally, the thinner the sheet, the greater the difference in phase velocity between the S0 and A0 waves within the 0 to 10 MHz range.

Figure 4a shows the phase velocity of S0 and A0 waves in a 0.01 mm nickel foil within the 0 to 10 MHz range. At this thickness, the cutoff frequency *f_C-A1_* = 148.2 MHz, which lies beyond the frequency range depicted in Figure 4a. The phase velocity of the S0-mode Lamb wave is significantly higher than that of the A0 mode, and within a certain frequency range, its phase velocity remains relatively stable. For instance, between 0 and 10 MHz, the phase velocity decreases by only 0.1191‰. In contrast, the A0 mode exhibits a larger relative change in phase velocity, resulting in poorer stability and reliability compared to the S0 mode.

In this scenario, the S0 wave propagates in the x-direction of the sheet, with particle vibration aligned parallel to the propagation direction. This behavior within the sheet can be considered analogous to that of a traditional longitudinal wave. For ultra-thin ferromagnetic metal films, the S0 wave may even be described as ‘longitudinal wave-like’.

It is important to emphasize that the primary focus of this research and experiment is on the S0 wave. While the S0 wave is dominant in the experiment, the A0 wave’s simultaneous presence causes some interference. As shown in the dispersion curves, for a nickel foil with a thickness of 0.2 mm, the velocity of the S0 wave is more than double that of the A0 wave when the frequency is below *f_C-A1_*. For a 10 micron nickel foil, the S0 wave’s velocity remains significantly greater than that of the A0 wave. In even thinner micro or nanometer-scale nickel films or foils, although both wave modes exist simultaneously, the A0 wave’s interference with the S0 wave becomes relatively insignificant when analyzed in the time domain.

## 3. Experiment & Result Discussion

### 3.1. Experiment Design

**Figure 5:**
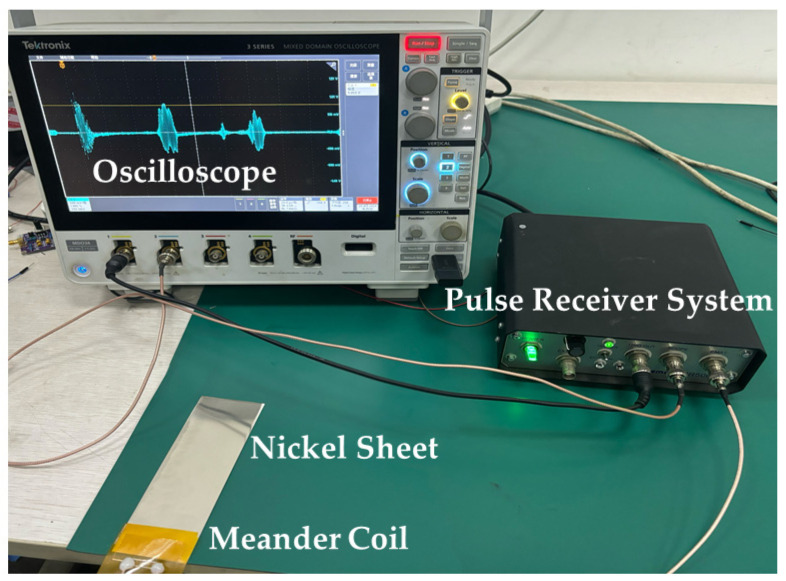
Actual configuration of the experimental setup.

The experimental design involves a single meander coil for both generation and reception of Lamb waves in nickel sheets. As illustrated in Figure 5, the experimental setup includes an oscilloscope (Tektronix, Beaverton, OR, USA), a pulse-receiver system (Sonemat PR-5000), a double-layer meander coil with a wire spacing of 2 mm, nickel sheets, and connecting wires. The PR system (Sonemat PR-5000) provides a spike driving current pulse with a width of 100 ns at 450 V to the meander coil, and wideband low-noise signal amplification for detection.

**Figure 6:**
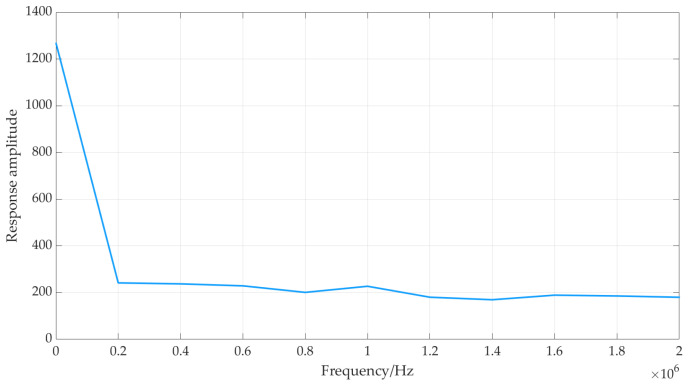
FFT of an excitation signal from Sonemat pr-5000 obtained after connecting the coil.

From Figure 6, it can be observed that there is a significant response amplitude at a frequency of 0, indicating the presence of a DC component in the excitation signal. The magnetic field maintained by the DC component over a specific time period is referred to as a “relatively static magnetic field”, while the rapid changes induced by the AC component generate a “relatively dynamic magnetic field”. It is important to note that the terms “static” and “dynamic” magnetic fields here are relative to the duration of the current rather than absolute.

In the actual experiment, the double-layer meander coil is used to transmit and receive signals, ensuring optimal signal strength. The dispersion curves analyzed in Section 2.2 demonstrate that the Lamb wave velocity generated by the coil is primarily influenced by the sheet thickness and frequency. The velocity experiments, as depicted in Figure 5, were conducted using three different sizes of nickel sheets at a thickness of 0.2 mm, as detailed in Table 1. The ultrasonic flight distance is defined as twice the length between the center of the meander coil and the far edge of the nickel sheet. The difference in ultrasonic flight distance between nickel sheets of various lengths is either 100 mm or 200 mm. Since S0-mode Lamb waves propagate at a constant velocity in nickel sheets of the same thickness, the speed of sound can be calculated by dividing the difference in flight distance by the difference in time.

**Figure 7:**
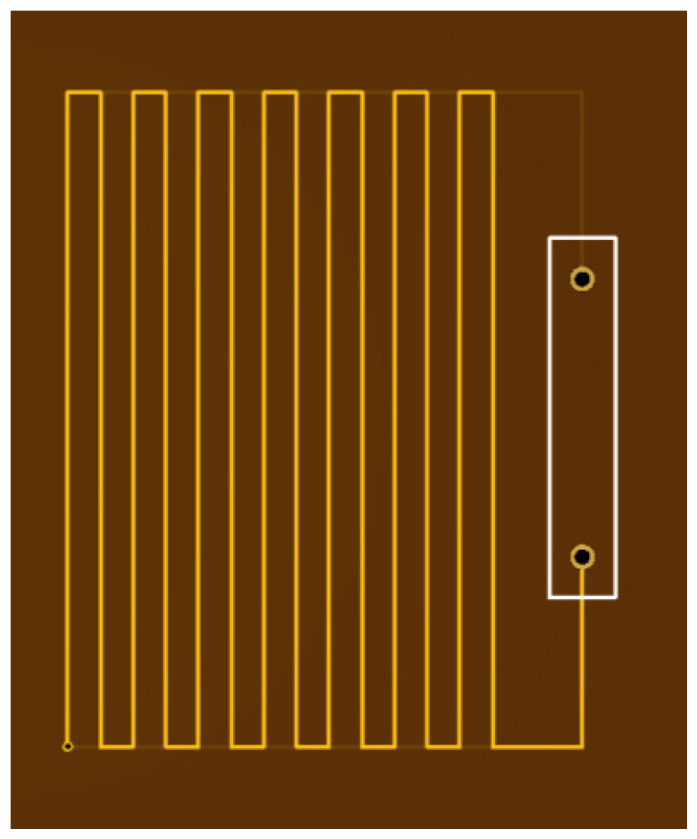
Double-layer meander coil with 2 mm wire spacing.

As shown in Figure 7, the coils used in this experiment are double-layer meander coils with a wire spacing of 2 mm. The coils were positioned as depicted in Figure 5, with an overall width of 50 mm, including the transparent plastic layer. The length of the coil parallel to the y-direction was 40 mm.

**Figure 8:**
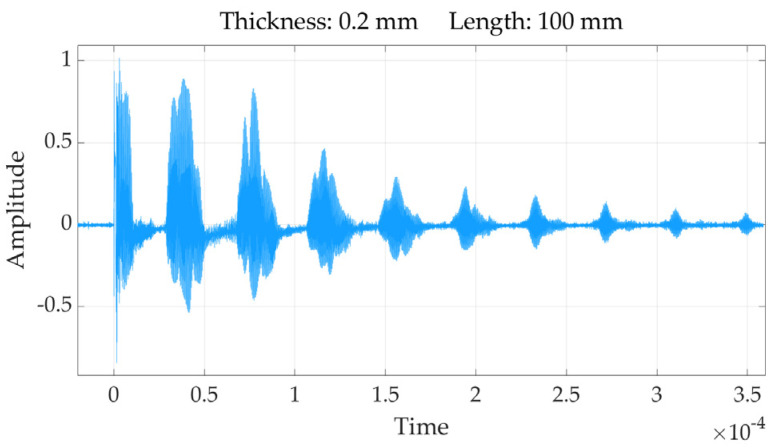
The a−time domain signal of experimental group 1.

We measured the meander coil at 2.625 MHz using the HIOKI IM 3536 impedance analyzer. The results are as follows: the impedance is 31.92 ohms, the inductance is 1.87 μH, the phase angle is 75°, and the resistance is 8.24 ohms. It should be acknowledged that the meander coil utilized in this experiment was not the optimal choice; however, it still yielded satisfactory experimental results. Originally, we intended to use a through-type helical coil. Yet, given the substantial difficulties in stabilizing this configuration within our setup, we ultimately decided against it. Had the helical coil been employed, a single, well-defined peak would likely have been observed in the ultrasonic echo rather than the three peaks obtained with the meander coil. For instance, using a nickel sheet with a thickness of 0.2 mm and length of 100 mm, the results are presented in Figure 8 in the following section. While three peaks were noted in the echo due to the nature of the meander coil, the most prominent peak can be selected as a reference point for calculations. The subsequent sections will provide a comprehensive elucidation of the experimental methods and findings.

### 3.2. Results of the Experiment

#### 3.2.1. Ultrasonic Velocity of Sound Measurement

In the course of the experiments, three repetitions were conducted on nickel sheets with a thickness of 0.2 mm and lengths of 100 mm, 150 mm, and 200 mm. For example, the time–domain signal obtained from the experiment on the 0.2 mm thick, 100 mm long, and 50 mm wide nickel sheet is presented in Figure 8. It is evident that there are ten peaks discernible in this figure. Peaks exceeding 25% of the maximum peak value were considered valid, yielding a total of six valid peaks. The time interval between two peaks represents the ultrasonic wave’s flight time from the coil’s center to the far edge of the nickel sheet, which is recorded as completing one round trip.

The coordinates of the valid echo peaks, as shown in Table 2, should be recorded in terms of the *x*-axis time of peak (ToP, measured in ×10^−5^ s). The average times between peaks were calculated and are noted in the table.

The speed of S0 can be calculated using the following equation:

(4) $$c=\frac{2△L}{△t}$$

The symbol △*L* represents the difference in the actual lengths of the two nickel sheets, while △*t* denotes the time interval between two peaks in the time–domain signal, measured from nickel sheets of varying lengths. Finally, the symbol *c* represents the actual ultrasonic velocity, as determined from the measurements.

In the experimental design section above, the lengths of the nickel sheets were designed as 100 mm, 150 mm, and 200 mm. However, the actual lengths of the three nickel sheets used were measured as 100 mm, 153 mm, and 202 mm using a vernier caliper. Furthermore, to ensure the consistency of the measured flight distances in the actual measurement process, the coils were positioned at both ends of each nickel sheet during the experiments.

#### 3.2.2. Computational Analysis of Experimental Results

The average time intervals between the time–domain peaks for the three different nickel sheet lengths were 3.8928, 5.8982, and 7.9818 (×10^−5^ s), respectively, across nine experimental trials. These values were used to calculate the mean actual velocity of the S0-mode Lamb wave in the nickel sheet, as shown in Table 3. According to the dispersion curve in Figure 4, at a frequency of 2.625 MHz, the theoretical speed of the S0-mode Lamb wave in a 0.2 mm nickel sheet is 5.0195 m/ms. Table 3 reveals that the actual measured speeds of the S0-mode Lamb wave in the nickel sheet differ from the theoretical value by 5.3033%, 6.2974%, and 0.6076%, respectively.

**Figure 9:**
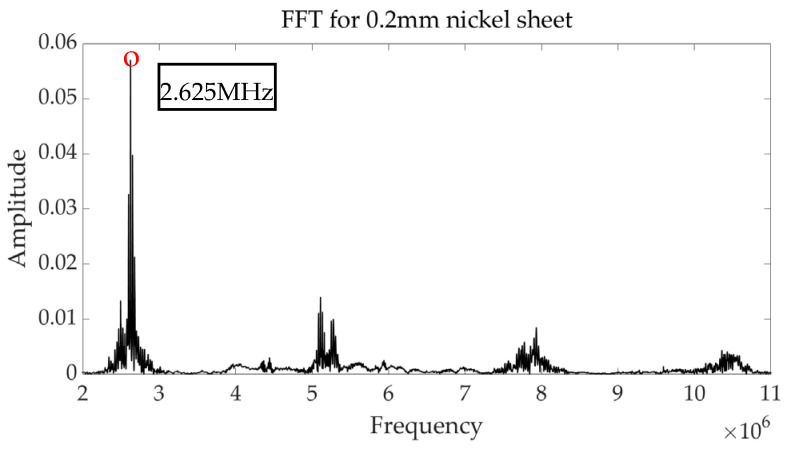
FFT for the 0.2 mm nickel sheet: Results from experiment 1.

Referring to the dispersion curves shown in Figure 4 of Section 2.2, it can be observed that only two Lamb wave modes, S0 and A0, are present at frequencies below 7.410 MHz for a 0.2 mm sheet thickness. The time–domain signals obtained from the nine experimental trials on the 0.2 mm nickel sheet were converted into frequency–domain signals using fast Fourier transform (FFT). As a case in point shown in Figure 9, the ultrasonic frequencies from the experimental group 1 with nickel sheet lengths at 100 mm exhibit notable consistency, with a concentration around 2.625 MHz. This experimental concentration frequency is lower than the cutoff frequency of 7.410 MHz, indicating that the time interval between peaks represents the round-trip flight time of the S0 wave from the center of the coil to the far edge of the nickel sheet. Furthermore, the frequency–domain signal exhibits a strong concentration of frequencies along with the presence of harmonics.

It is evident from Table 3 that the experimentally derived sound velocities of the S0-mode Lamb wave deviate from the theoretical values. To further analyze these discrepancies, the nine sets of experimental data were subjected to orthogonal analysis. From the aforementioned nine sets of experimental results, two sets of experimental results, featuring nickel sheets of different lengths, were selected for comparative calculations using the specified formula. The results of these calculations are presented in Table 4. It is important to clarify that the first column of Table 4 represents the comparison between two sets of experimental results. For example, the “1–4” in the first row indicates the differences between the measurements obtained from the first group using a 100 mm nickel sheet and the fourth group using a 153 mm nickel sheet. In this case, △L represents the length difference between the two nickel sheets, which is 53 mm; △t denotes the absolute value of the difference in the average time intervals obtained from the two sets of experiments; and c is the actual sound speed calculated using Equation (4).

**Figure 10:**
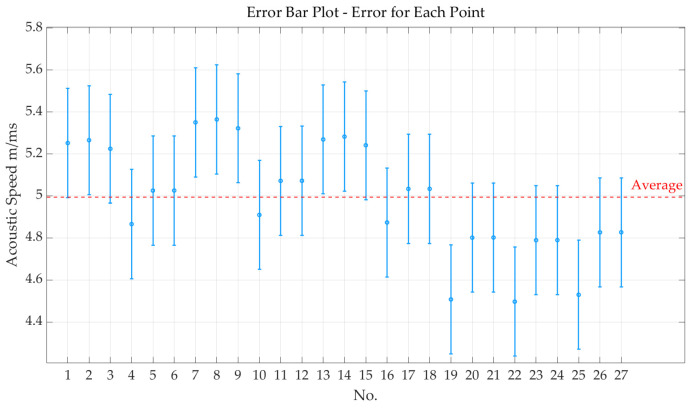
Error bars for the 27 data sets.

The aforementioned 27 groups were subjected to analysis, resulting in an average sound speed of 4.9946 m/ms. We plotted error bars in Figure 10 for the 27 data sets mentioned above, clearly illustrating the mean and corresponding error range for each set. The error bar visualization provides an intuitive way to observe the variability within each group and between different groups, helping us assess the stability and reliability of the experimental results. This type of graph not only aids in understanding the central tendency of the data but also effectively highlights differences and uncertainties across the various data sets.

Table 5 lists the phase and group velocities determined from a dispersion diagram of a 0.2 mm thickness nickel sheet at a frequency of 2.625 MHz. The actual phase velocity shows a 0.4985% discrepancy compared to the theoretical value of 5.0195 m/ms, indicating a high level of reliability.

## 4. Conclusions and Future Works

Experimental verification confirmed that a stable S0-mode Lamb wave could be generated in nickel sheets using a single coil driven by a composite current. By applying a composite signal with both DC and AC components through a double-layer meander coil placed on a 0.2 mm nickel sheet, an S0-mode Lamb wave at a frequency of 2.625 MHz was successfully excited. The velocity of this Lamb wave, measured experimentally, was 4.9946 m/s, demonstrating high accuracy with only a 0.4985% deviation from the theoretical value.

In future work, magnetostrictive materials will be explored as the sound-generating element in ultrasonic transducers, functioning similarly to piezoelectric wafers in piezoelectric transducers. While challenging, this approach has the potential to open up new applications in biomedical sensing and structural health monitoring.

## Figures and Tables

**Table 1:** Parameters of the nickel sheet used in the experiment *.

No.	Thickness (mm)	Length (mm)
1; 2; 3	0.2	100
4; 5; 6	0.2	150
7; 8; 9	0.2	200

* The nickel sheets used in this experiment were all 50 mm in width.

**Table 2:** Results from the time–domain signal, times of peaks (×10^−5^ s).

Length	No.	ToP 1	ToP 2	ToP 3	ToP 4	ToP 5	ToP 6	Ave. Time Interval
100 mm	1	3.8828	7.7274	11.652	15.614	19.427	23.274	3.8782
	2	3.8422	7.7247	11.646	15.529	19.377	23.263	3.9154
	3	3.8422	7.7244	11.606	15.455	19.379	23.266	3.8847
153 mm	4	5.7022	11.599	17.611	23.506	29.325	35.184	5.8964
	5	5.6990	11.709	17.601	23.478	29.335	35.155	5.8912
	6	5.6222	11.593	17.602	23.459	29.337	35.157	5.9070
202 mm	7	7.3573	15.377	23.781	31.569	/	/	8.0706
	8	7.8166	15.779	23.727	31.629	/	/	7.9375
	9	7.8191	15.758	23.766	31.631	/	/	7.9373

**Table 3:** The estimated values of S0 velocity in the nickel sheet.

△L (×10^−3^ m)	△t (×10^−2^ ms)	c (m/ms)
53	2.0054	5.2857
49	2.0836	4.7034
102	4.0890	4.9890

**Table 4:** Orthogonal analysis—actual velocity of sound in a nickel sheet for Lamb waves of mode S0.

No.	△L (×10^−3^ m)	△t (×10^−2^ ms)	c (m/ms)
1–4	53	2.0182	5.2522
1–5	53	2.0130	5.2658
1–6	53	2.0288	5.2248
1–7	102	4.1924	4.8659
1–8	102	4.0593	5.0255
1–9	102	4.0591	5.0257
2–4	53	1.9810	5.3508
2–5	53	1.9758	5.3649
2–6	53	1.9916	5.3224
2–7	102	4.1552	4.9095
2–8	102	4.0221	5.0720
2–9	102	4.0219	5.0722
3–4	53	2.0117	5.2692
3–5	53	2.0065	5.2828
3–6	53	2.0223	5.2416
3–7	102	4.1859	4.8735
3–8	102	4.0528	5.0336
3–9	102	4.0526	5.0338
4–7	49	2.1742	4.5074
4–8	49	2.0411	4.8013
4–9	49	2.0409	4.8018
5–7	49	2.1794	4.4967
5–8	49	2.0463	4.7891
5–9	49	2.0461	4.7896
6–7	49	2.1636	4.5295
6–8	49	2.0305	4.8264
6–9	49	2.0303	4.8269

**Table 5:** The phase and group velocities were determined from a dispersion diagram of a 0.2 mm thickness nickel sheet at a frequency of 2.625 MHz.

Guided Waves Mode	Phase Velocity m/ms	Group Velocity m/ms
S0	5.0195	4.9802
A0	1.8336	2.7909
S1	/	/
A1	/	/

## Data Availability

The authors confirm that the data supporting the findings of this study are available within the article.

## References

[1] Guo X., Zhu W., Qiu X., Xiang Y. (2022). A Lorentz Force EMAT Design with Racetrack Coil and Periodic Permanent Magnets for Selective Enhancement of Ultrasonic Lamb Wave Generation. Sensors.

[2] Del Moral A. (2007). Magnetostriction and Magnetoelasticity Theory: A Modern View. Handbook of Magnetism and Advanced Magnetic Materials.

[3] Murayama R. (1996). Driving Mechanism on Magnetostrictive Type Electromagnetic Acoustic Transducer for Symmetrical Vertical-Mode Lamb Wave and for Shear Horizontal-Mode Plate Wave. Ultrasonics.

[4] Clarke T., Cawley P., Wilcox P.D., Croxford A.J. (2009). Evaluation of the Damage Detection Capability of a Sparse-Array Guided-Wave SHM System Applied to a Complex Structure under Varying Thermal Conditions. IEEE Trans. Ultrason. Ferroelectr. Freq. Control.

[5] Lee J.K., Kim H.W., Kim Y.Y. (2013). Omnidirectional Lamb Waves by Axisymmetrically-Configured Magnetostrictive Patch Transducer. IEEE Trans. Ultrason. Ferroelectr. Freq. Control.

[6] Sun W., Liu G., Xia H., Xia Z. (2018). A Modified Design of the Omnidirectional EMAT for Antisymmetric Lamb Wave Generation. Sens. Actuators A Phys..

[7] Liu Z., Zhong X., Xie M., Liu X., He C., Wu B. (2017). Damage Imaging in Composite Plate by Using Double-Turn Coil Omnidirectional Shear-Horizontal Wave Magnetostrictive Patch Transducer Array. Adv. Compos. Mater..

[8] Murayama R. (1999). Study of Driving Mechanism on Electromagnetic Acoustic Transducer for Lamb Wave Using Magnetostrictive Effect and Application in Drawability Evaluation of Thin Steel Sheets. Ultrasonics.

[9] Wang K., Zhang R., Feng B., Guo Y., Kang Y., Song Y., Ma H. Magnetostrictive Ultrasonic Torsional Wave Detection Method for High-Density Polyethylene Pipe Weld Status. Proceedings of the 2024 IEEE International Instrumentation and Measurement Technology Conference (I2MTC).

[10] Rueter D. (2016). Induction Coil as a Non-Contacting Ultrasound Transmitter and Detector: Modeling of Magnetic Fields for Improving the Performance. Ultrasonics.

[11] Rueter D. (2017). Experimental Demonstration and Circuitry for a Very Compact Coil-Only Pulse Echo EMAT. Sensors.

[12] Park C.I., Kim W., Cho S.H., Kim Y.Y. (2005). Surface-Detached V-Shaped Yoke of Obliquely Bonded Magnetostrictive Strips for High Transduction of Ultrasonic Torsional Waves. Appl. Phys. Lett..

[13] Isla J., Cegla F. (2017). EMAT Phased Array: A Feasibility Study of Surface Crack Detection. Ultrasonics.

[14] Wang J., Zheng Z., Chan J., Yeow J.T.W. (2020). Capacitive Micromachined Ultrasound Transducers for Intravascular Ultrasound Imaging. Microsyst. Nanoeng..

[15] Birjis Y., Swaminathan S., Nazemi H., Raj G.C.A., Munirathinam P., Abu-Libdeh A., Emadi A. (2022). Piezoelectric Micromachined Ultrasonic Transducers (PMUTs): Performance Metrics, Advancements, and Applications. Sensors.

[16] Zang J., Fan Z., Li P., Duan X., Wu C., Cui D., Xue C. (2022). Design and Fabrication of High-Frequency Piezoelectric Micromachined Ultrasonic Transducer Based on an AlN Thin Film. Micromachines.

[17] Gandomzadeh D., Abbaspour-Fard M.H. (2020). Numerical Study of the Effect of Core Geometry on the Performance of a Magnetostrictive Transducer. J. Magn. Magn. Mater..

[18] Teh K.-S., Cheng Y.-T., Lin L. (2005). MEMS Fabrication Based on Nickel-Nanocomposite: Film Deposition and Characterization. J. Micromech. Microeng..

[19] Weber N., Zappe H., Seifert A. (2012). An All-Nickel Magnetostatic MEMS Scanner. J. Micromech. Microeng..

[20] Sun S., Dai X., Sun Y., Xiang X., Ding G., Zhao X. (2017). MEMS-Based Wide-Bandwidth Electromagnetic Energy Harvester with Electroplated Nickel Structure. J. Micromech. Microeng..

[21] Rose J.L. (2014). Ultrasonic Guided Waves in Solid Media.

